# CT for Cervical Spine Clearance in the Obtunded Adult Blunt Trauma Patient is Appropriate in a Resource‐Constrained Environment

**DOI:** 10.1002/wjs.70386

**Published:** 2026-05-08

**Authors:** Reuben He, Victor Kong, Jonathan Ko, William Yeung, Daniel Lee, Nikilesh Babu, Joshua Ahn, Howard Wain, Grant Laing, Damian Clarke

**Affiliations:** ^1^ Department of Surgery University of Auckland Auckland New Zealand; ^2^ Department of Surgery University of KwaZulu‐Natal Durban South Africa; ^3^ Trauma Unit Chris Hani Baragwanath Hospital Johannesburg South Africa; ^4^ Department of Surgery University of the Witwatersrand Johannesburg South Africa

**Keywords:** blunt trauma, cervical spine, clearance, obtunded, orthopaedics

## Abstract

**Purpose:**

Cervical spinal injury (CSI) following blunt trauma to the neck can have devastating consequences. There is a current controversy surrounding how CSIs are excluded in obtunded patients. This study aimed to evaluate the utility of CT in cervical spine clearance in a high‐volume trauma center in a developing world setting.

**Methods:**

Longitudinal 11‐year data (2012–2022) were collected from the Hybrid Electronic Medical Registry (HEMR), based at the Pietermaritzburg Metropolitan Trauma Service, to identify all obtunded patients (GCS < 15) who underwent a cervical CT scan. The accuracy of CT imaging in identifying CSI was evaluated by calculating the diagnostic sensitivity, specificity, negative predictive value, and positive predictive value against the final admission diagnosis.

**Results:**

In total, 1039 obtunded blunt trauma patients underwent CT of the C‐spine. 121 (12%) of these patients had a CSI demonstrated on CT or MRI. Six (5%) required surgery, and 115 (95%) were treated nonoperatively. In total, 113 (93%) of these patients had a positive CT, and 8 (7%) patients had a false‐negative CT but went on to have a positive MRI for CSI; none of these patients with an injury identified on MRI required surgery. CT demonstrated a sensitivity and specificity of 93.4% and 96.2%, with a negative predictive value of 99.1% for identifying CSI.

**Conclusion:**

In the obtunded trauma patient, CT correctly identifies all patients with a bony fracture of the cervical spine who will require intervention. In a small subset of patients, CT will miss non‐bony injuries. MRI may be necessary for further investigation in the context of persisting clinical concern for CSI (e.g., neurological deficit, polytrauma, unstable vital signs (especially hypotension), and intubation). Most of these non‐bony injuries do not require surgery.

## Graphical abstract

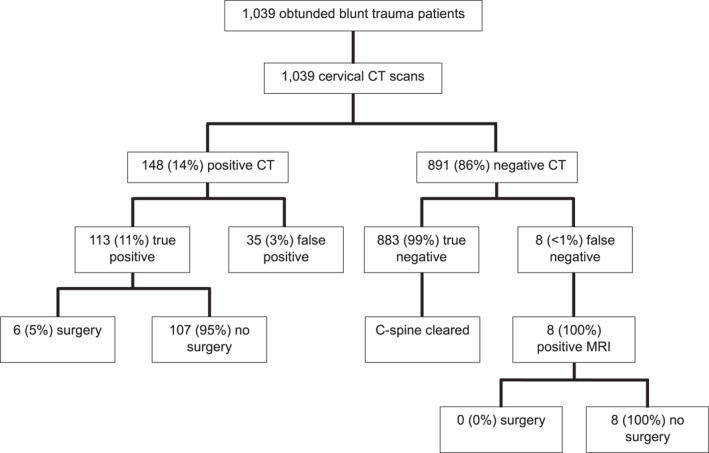

## 1 Introduction

This study aimed to review the utility of CT in cervical spine clearance in a high‐volume trauma center in a developing‐world setting.

Cervical spinal injury (CSI) following blunt trauma to the neck can have devastating consequences. Therefore, clinicians must be vigilant in diagnosing such an injury and protecting patients with potential CSI. Excessive movement may exacerbate a CSI, and a delayed diagnosis of such an injury places the patient at increased risk. Blunt neck trauma often occurs in polytrauma settings involving diverse body regions. These injuries may be immediately life‐threatening, and their management may be prioritized over CSI.

Many blunt polytrauma patients have an impaired level of consciousness or are intubated, thus precluding adequate clinical assessment. In response to this, the Advanced Trauma Life Support (ATLS) course teaches that cervical spine immobilization must be maintained throughout the patient's management until a CSI has been excluded [1]. Recent studies have re‐assessed the question of whether high‐quality computed tomography (CT) alone is sufficient to exclude CSI, with some studies advocating for further magnetic resonance imaging (MRI) and others concluding that additional MRI may increase healthcare resource use without revealing significant injuries [2, 3, 4]. Therefore, there remains controversy surrounding how an injury is excluded and the cervical spine “cleared” [2].

Prior to the advent of the CT scan, great emphasis was placed on detailed plain radiographs of the entire cervical spine, including through‐the‐mouth views and often so‐called swimmers' views of the seventh cervical spine body. Obtaining these images can be challenging, and artefacts and patient‐related issues often complicate the process of obtaining adequate images. High‐quality multi‐detector CT scan has made these plain radiographs obsolete, and it is relatively easy to obtain detailed images of the cervical spine. The current issue is that the CT scan, although providing particularly good images of the bony structures of the neck, cannot delineate the cord structures. There is concern that non‐bony injury to the ligaments of the neck or the spinal cord itself may not be detected. Some authors have suggested that MRI may be required to exclude these non‐bony injuries [2, 5]. MRI is not readily available and can be challenging to obtain in an obtunded, ventilated trauma patient for logistical reasons. Recent literature has suggested that although high‐quality CT can exclude clinically significant CSI with high sensitivity, there remains a small but clinically significant incidence of missed injuries in patients with an abnormal neurologic examination prompting imaging. In these cases, further imaging with MRI is warranted [4, 6, 7].

Compounding this debate is the reality that in many resource‐constrained clinical environments, MRI is not readily available and can be challenging to obtain in an obtunded, ventilated trauma patient for logistical reasons. This has forced clinicians working in such settings to adopt a selective approach. This study was conducted in a high‐volume trauma center in a developing world setting, where resource constraints have historically encouraged a highly selective clinical approach to all aspects of trauma care. It is hoped that the data from this project will provide high‐quality evidence to support these local policies and contribute to the international debate on this topic.

## 2 Materials and Methods

### 2.1 Clinical Setting

The Pietermaritzburg Metropolitan Trauma Service (PMTS) is based at Grey's Hospital in Pietermaritzburg, South Africa. The PMTS provides definitive trauma care to Pietermaritzburg and its surrounding catchment area, which has a total population of over four million people. It is one of the largest academic trauma centers in the region, with over 4000 trauma admissions annually, of which over half are secondary to non‐penetrating injuries. The Hybrid Electronic Medical Registry (HEMR) is a regional electronic trauma database that captures all trauma admissions to Grey's Hospital.

### 2.2 Management Protocol

**FIGURE 1:**
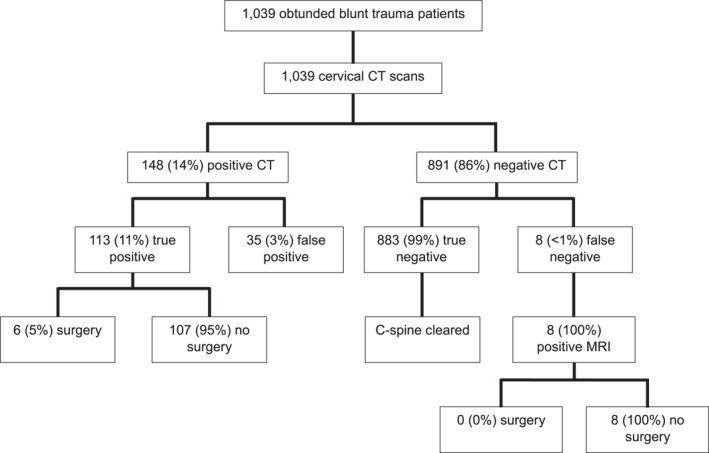
Obtunded blunt trauma patients between 2012 and 2022. C‐spine, cervical spine; CSI, cervical spine injury; CT, computed tomography; MRI, magnetic resonance imaging.

The trauma surgeons at our institution lead the initial assessment and evaluation. They resuscitate and investigate the patients before involving the required sub‐disciplines (in this case, spinal injury is managed by orthopaedics or neurosurgery based on a roster system). All blunt trauma patients are reviewed according to the NEXUS criteria for C‐spine imaging. Failure to meet the NEXUS criteria will result in CT head and neck imaging to investigate for possible CSI. Our institutional protocol utilizes non‐contrast enhanced CT, unless there is concern for vascular injury, such as symptoms of (posterior circulation) ischaemia or high‐risk fracture patterns (e.g., fracture extending into transverse foramen, hyperextension/rotation injury). In our study, an obtunded patient was defined as a patient with a non‐normal Glasgow coma scale (GCS) score, altered sensation, intubation, or unreliable exam. A cutoff of GCS ≤ 14 was used for this study based on historical studies [8, 9, 10, 11]. By definition, none of these patients meet the NEXUS criteria, so CT imaging is completed for all of them. Patients with CSI identified on CT scan are referred to neurosurgery and/or orthopaedic surgery for appropriate management, depending on the injury. Patients with normal CT scans are cleared from having CSI after review by orthopaedics and neurosurgery, based on a spinal roster. Further review of the medical record failed to reveal any “bounce backs” with significant spinal injuries missed on the initial evaluation. In patients with normal CT scans, those with persisting clinical concern for CSI (e.g., neurological deficit, abnormal reflex testing, polytrauma, unstable vital signs (prolonged hypotension), intubation) undergo an MRI scan to further investigate possible CSI. A positive MRI scan was defined as a scan with an abnormal finding. A clinically significant injury on MRI was defined as an abnormal finding that could account for focal neurological deficits. An algorithm for this management protocol, with the patient numbers from our study, is demonstrated in Figure 1.

### 2.3 The Study

In this single‐center retrospective study, longitudinal 11‐year data (2012–2022) were collected from the HEMR trauma database to identify all blunt trauma patients who failed the NEXUS low‐risk criteria over the study period [12]. Of these patients, all obtunded (Glasgow coma scale (GCS) < 15) patients who underwent a CT scan of the C‐spine were included in the study. Patients with a GCS of 15 or those with missing data were excluded from the analysis. Data were extracted into a pro forma Google spreadsheet by a single investigator (RH) and independently validated by a second investigator (VK). Data extraction followed established guidelines to minimize bias [13]. Data comprised clinical information from assessment to discharge, including presenting complaint, vital signs, lab results, investigations, diagnosis/injuries, clinical course, operation notes, and post‐operative recovery. The primary study outcome was CSI based on radiological imaging, with a positive finding defined as any injury to the C‐spine, for example, fracture, haematoma, ligamentous injury, etc. Ethics approval for maintaining our registry and conducting this study was formally obtained from the Biomedical Research Ethics Committee of the University of KwaZulu‐Natal (BCA 221/13). Data were extracted up to 2022; subsequent analysis was completed in 2025 due to clinical commitments.

### 2.4 Statistical Analysis

This study sought to investigate the accuracy of CT scans for the detection of clinically significant CSI after blunt trauma. This was achieved by evaluating the diagnostic sensitivity, specificity, negative predictive value (NPV), positive predictive value (PPV), false positive rate, and false negative rate against a final diagnosis of CSI, which was a result of the accumulation of all investigations and/or procedures throughout the patient's hospital admission. Descriptive analysis was conducted to summarize the raw data by calculating median values for continuous variables and frequencies/proportions for categorical variables. Tables and figures were used to present the data. Additionally, the data were stratified based on those with CSI and those without. In univariate analysis, categorical outcomes were compared using the chi‐squared test, and continuous variables were compared using the independent‐samples Student's t‐test or the Mann–Whitney *U* test, as appropriate. Variables with a *p* value < 0.15 were then entered into a multivariate logistic regression analysis to identify independent variables associated with CSI. Two‐tailed *p*‐values < 0.05 were considered statistically significant. Statistical analysis was performed using SPSS (version 30, IBM Corp, Armonk, NY, USA).

## 3 Results

### 3.1 Overview

During the study period, 1039 obtunded blunt trauma patients were deemed not suitable for the NEXUS assessment and underwent a CT scan of the C‐spine. There were 811 (78%) males and 228 (22%) females. The median age was 30 (interquartile range (IQR) 22–39) years, and the median injury severity score (ISS) was 13 (IQR 9–17). The median GCS score on admission was 12 (IQR 7–14). Table 1 describes the characteristics of these blunt trauma C‐spine patients. The most common mechanisms of blunt trauma, in descending order, were motor vehicle accident (35%), assault (29%), pedestrian vehicle accident (21%), falls from height or ground level (11%), crush injury (1%), and animal‐related injury (< 1%). Other mechanisms (e.g., hanging) accounted for the remaining 3%. All patients underwent a CT scan of the C‐spine, and 94 (9%) underwent an MRI. The median time from injury to hospital admission was one day (IQR 0–1). The mortality rate was 6%, but none of the deaths was directly related to CSI. Figure 1 details the management algorithm of blunt trauma patients in this study.

**TABLE 1:** Characteristics of blunt trauma C‐spine patients.

Variable	Total (*n* = 1039)
Median age (IQR)	30 (22–39)
Male	811 (78%)
Blunt trauma mechanism	
MVA	367 (35%)
PVA	221 (21%)
Fall	111 (11%)
Assault	301 (29%)
Crush	8 (1%)
Animal related	4 (< 1%)
Other	27 (3%)
Median ISS (IQR)	13 (9–17)
Median head/neck AIS (IQR)	3 (2–3)
Median face AIS (IQR)	2 (1–2)
Median chest AIS (IQR)	2 (2–3)
Median abdomen AIS (IQR)	3 (2–3)
Median GCS on admission (IQR)	12 (7–14)
Intubated	211 (20%)
Midline C‐spine tenderness	371 (36%)
Focal neurological deficit	48 (5%)
Unevaluable	316 (30%)
Imaging	
CT	1039 (100%)
MRI	94 (9%)

*Note:* Mean GCS: [10, e.g., based on median 12 and IQR 7–14, assuming skewness].

Abbreviations: AIS, abbreviated injury scale; C‐spine, cervical spine; CT, computed tomography; GCS, Glasgow coma scale; IQR, interquartile range; ISS, injury severity score; MRI, magnetic resonance imaging; MVA, motor vehicle accident; PVA, pedestrian vehicle accident.

### 3.2 Injuries and Management

Overall, 121 (12%) of the 1039 patients had a CSI demonstrated on CT or MRI. Table 2 details these injuries and their subsequent management. Of these 121 patients, 6 (5%) ultimately required surgery, and 115 (95%) were treated nonoperatively. Apart from a hard collar, non‐surgical management of patients with CSI involved a combination of alternative cervical‐thoracic orthotic placement, analgesia, and physical therapy. In total, 113 (93%) patients with CSI were identified on CT scans, and 8 (7%) were demonstrated on MRI scans. All eight patients had negative CT but positive MRI after concerns regarding a persisting neurological deficit. Of these eight patients with false‐negative CT scans, 6 (75%) had C‐spine immobilization until their MRI scan. In contrast, the remaining 2 (25%) did not have C‐spine immobilization due to risks of increased intracranial pressure in the context of traumatic brain injury. On subgroup analysis, the MRI findings in these patients consisted of spinal cord injury (*n* = 5), stable minor ligamentous injury (*n* = 2), and subtle C7 transverse process fracture (*n* = 1). Of the five patients with spinal cord injury, four were American Spinal Injury Association (ASIA) Impairment Scale Grade D, and one was ASIA scale Grade C. The spinal cord injuries were consistent with neurological deficits. The remaining patients in this subgroup had minor peripheral deficits (e.g., isolated weakness of wrist extension). All eight had persisting clinical concern (e.g., focal deficits) per protocol, confirming selective MRI use. None of these patients with MRI‐identified injuries required surgical intervention. All six patients who required surgery had a bony fracture demonstrated on CT scan. The surgical procedures required included fixation, fusion, or arthrodesis (*n* = 5) or laminectomy and decompression (*n* = 1). The AO spine classifications of patients who required surgery are outlined in Table 3. The most common CSI patterns, in descending order, were fracture (95%), subluxation or dislocation (9%), and subdural haematoma (1%). Some patients had multiple injuries to the cervical spine (e.g., multilevel injury, or more than one type of injury). The levels of CSI, in descending order, were C7 (41%), C2 (31%), C1 (29%), C6 (27%), C5 (26%), C4 (18%), and C3 (11%). The most common level of injury requiring operative intervention was C2 (*n* = 2). In total, 778 (75%) patients were managed with a hard collar during the diagnostic period. Of these patients, 657 (84%) had normal CT imaging.

**TABLE 2:** Injuries and management of patients with CSI.

	CSI
Variable	Total (*n* = 121)
CSI	121 (100%)
Type of injury	
Fracture	115 (95%)
Subluxation/dislocation	11 (9%)
Subdural haematoma	1 (1%)
Level of injury	
C1	35 (29%)
C2	37 (31%)
C3	13 (11%)
C4	22 (18%)
C5	32 (26%)
C6	33 (27%)
C7	49 (41%)
Treatment	
Non‐surgical	115 (95%)
Hard collar	94 (78%)
Surgical	6 (5%)
Fusion/fixation/arthrodesis	5 (4%)
Decompression/laminectomy	1 (1%)

*Note:* Percentages may exceed 100% due to multiple injuries per patient.

Abbreviation: CSI, cervical spine injury.

**TABLE 3:** AO spine classification of patients that required surgery.

	Surgery
AO spine classification system	Total (*n* = 6)
Type C	4 (66%)
Fusion/fixation/arthrodesis	4
Type B2	2 (33%)
Fusion/fixation/arthrodesis	1
Decompression/laminectomy	1

### 3.3 Predictors of CSI

In univariate analysis, there was no significant evidence of correlation between mechanism of injury and CSI (*p* > 0.05). Similarly, there was no significant evidence of correlation between admission GCS and CSI (*p* > 0.05). Abbreviated injury scale (AIS) for head/neck (*p* < 0.001) and chest (*p* < 0.001) was significantly associated with CSI. In contrast, other regions of the body (Table 1) had no statistical association (*p* > 0.05) with CSI. Patients who were unevaluable on admission (unable to complete clinical assessment reliably, e.g., intubated or sedated, severe pain, altered mental status, etc) were also significantly associated with CSI (*p* < 0.001). Focal neurological deficit or C‐spine tenderness was not significantly associated with CSI (*p* > 0.05). In multivariate analysis, only one variable was an independent predictor of CSI: patients who were unevaluable at admission (odds ratio (OR) 2.747, 95% confidence interval (CI) 1.870–4.036, *p* < 0.001).

### 3.4 CT Accuracy

CT demonstrated a sensitivity of 93.4% and a specificity of 96.2% when compared to the final diagnosis. The PPV was 76.4% and the NPV was 99.1%. Table 4 demonstrates the diagnostic accuracy of CT scan in detecting CSI in obtunded blunt trauma patients. The total number of positive CT scans was 148, which consisted of 113 true positives and 35 false positives. The false‐positive rate was 3.8% (35/918), and the false negative rate was 6.6% (8/121) for identifying CSI with CT. Of the 35 patients with false‐positive CT scans, the most common cause was motion artefact causing blurring or doubling of bone contours and distortion of adjacent soft tissues, leading to false appearances of acute fractures or non‐bony injuries. In these patients, 97% (*n* = 34) underwent a subsequent MRI to rule out CSI, with one patient experiencing resolution of clinical signs concerning CSI. False‐negative CT scans were primarily related to inferior soft tissue characterization of CT imaging compared to MRI. For all patients with CSI, either the CT scan of the spine identified an injury, or there was abnormal focal neurology on examination. This means that, when combined, CT and neurological examination were able to detect 100% of CSI in blunt trauma obtunded patients.

**TABLE 4:** Diagnostic accuracy of CT scan in detecting CSI.

		C‐spine injury		Predictive value
		Yes	No	
CT result	+ve	113	35	PPV $\frac{113}{148}$ = 76.4%
	−ve	8	883	NPV $\frac{883}{891}$ = 99.1%
Sensitivity & specificity		Sensitivity $\frac{113}{121}$ = 93.4%	Specificity $\frac{883}{918}$ = 96.2%	

Abbreviations: +ve, positive; −ve, negative; CSI, cervical spine injury; CT, computed tomography; NPV, negative predictive value; PPV, positive predictive value.

## 4 Discussion

This study presents a selective approach to imaging the cervical spine. Without the luxury of obtaining both CT scan and an MRI scan in all patients, our study provides a counterpoint to the more universal approach of CT and MRI for all obtunded patients. This selective approach is particularly relevant in resource‐constrained settings like ours, where MRI is limited to working hours, often delaying access by up to 48 h.

CSI can be devastating, and great emphasis is placed on preventing and limiting any exacerbation of such an injury by aggressively assessing and excluding a CSI. If a trauma patient is pain‐free and has no limitation of neck movement and no neurological deficit, then the chance of a CSI is highly remote. These asymptomatic and clinically alert patients are suitable for a clinical examination of the cervical spine. However, patients who cannot be assessed clinically because of obtundation or the presence of distracting or competing factors comprise a significant cohort of trauma patients. These compounding factors include associated life‐threatening injuries, intoxication, and a depressed level of consciousness. Our data revealed a 12% incidence of CSI in this group of patients; among patients with CSI, 5% require surgery. This suggests that current guidelines are appropriate, emphasizing and stressing the importance of in‐line cervical spine immobilization and aggressive exclusion of a CSI.

Given the potential consequences of CSI, the use of CT alone in cervical spine clearance has been controversial [2]. There is also a social dilemma of balancing the most cost‐effective method of clearing the C‐spine compared to the degree of error society is willing to tolerate. This dilemma has no clear answer, and decisions are left to local practitioners. The Eastern Association for the Surgery of Trauma (EAST) conditionally recommended cervical collar removal after a negative high‐quality C‐spine CT scan alone [6]. In the most extensive prospective studies by Inaba et al., it was found that the sensitivity of CT for ruling out clinically significant injury was 98.5%, whilst the negative predictive value was 99.97% [7]. Other authors have cautioned against reliance on CT scans and recommend more liberal use of MRI to improve CSI detection [2, 5]. However, liberal use of MRI is logistically challenging and often leads to delays in our environment. Instead, MRI should be performed on a case‐by‐case basis and is not routinely required in patients with no focal neurological deficit [14, 15]. Furthermore, Ertel et al. argued that adding MRI to the evaluation process was only cost‐effective if the risk of CSI exceeded 4% and this is especially relevant in our resource‐constrained setting [16].

In our study, 12% of obtunded blunt trauma patients ultimately had a CSI. In the current era, CT scan appears to be the most effective mechanism for assessing the cervical spine in obtunded polytrauma patients [14, 17, 18, 19, 20, 21]. Of the 121 patients with an injury, 113 (93%) were diagnosed on CT. The CT scan correctly identified all six patients who required spinal surgery. None of the remaining 8 (7%) patients with a positive MRI required surgical intervention. In these patients, an initial negative CT scan did not explain a persistent neurological deficit, and this prompted an MRI scan, which primarily identified spinal cord injury or clinically insignificant ligamentous injuries, none of which required surgery. This is consistent with other studies [22, 23]. In our environment, CT alone is sufficient in excluding clinically significant CSI. It should be noted that non‐operative management can present its own issues, with C‐collars causing problems such as pressure ulcers and agitation.

There are limitations to this study. Inclusion bias is present, as not all patients received complete imaging, including MRI. This could potentially lead to an underestimation of the frequency of spine injuries that would otherwise be detected on MRI. This must be counterbalanced against the fact that there were almost no clinical sequelae resulting from any potential missed injuries. The low rate of MRI scans may skew the sensitivity, specificity, PPV, and NPV calculations. Long‐term patient follow‐up is difficult to obtain using retrospective data. Therefore, our data does not capture consequential injuries that may only manifest over time (e.g., delayed presentation of spine instability), potentially underestimating missed injuries. Finally, our study is limited to data from a single center, which may restrict the external validity of this study to only centers with similar characteristics. This study presents a selective approach to imaging the cervical spine in a resource‐constrained clinical environment, where obtaining both a CT and an MRI for all patients is logistically challenging. Therefore, these findings may not be directly applicable to more resource‐abundant environments, where a universal approach of CT and MRI for all obtunded patients is possible. This selective approach is, however, particularly relevant to more resource‐constrained settings, where the majority of the world's trauma patients are treated and suggests that in these environments, a selective approach is safe and effective [24].

## 5 Conclusion

In the obtunded trauma patient, a cervical CT scan correctly identifies all patients with a bony fracture of the cervical spine who will require spinal fixation. A CT scan will not identify a soft tissue injury in a small subset of patients, and an MRI may be necessary to identify and delineate the injury. Most of these soft tissue injuries do not require surgery. A selective approach to imaging the cervical spine in obtunded trauma patients is reasonable and appropriate in resource‐constrained environments.

## Author Contributions

**Reuben He:** investigation, writing – original draft, methodology, writing – review and editing, software, formal analysis, data curation. **Victor Kong:** conceptualization, writing – original draft, writing – review and editing, supervision. **Jonathan Ko:** writing – review and editing. **William Yeung:** writing – review and editing. **Daniel Lee:** writing – review and editing. **Nikilesh Babu:** writing – review and editing. **Joshua Ahn:** writing – review and editing. **Howard Wain:** writing – review and editing. **Grant Laing:** writing – review and editing. **Damian Clarke:** supervision, conceptualization, writing – review and editing.

## Funding

The authors have nothing to report.

## Conflicts of Interest

The authors declare no conflicts of interest.

## Data Availability

Data sharing not applicable to this article as no datasets were generated or analyzed during the current study.

## References

[1] O. I. Schmidt , R. H. Gahr , A. Gosse , and C. E. Heyde , “ATLS(R) and Damage Control in Spine Trauma,” World Journal of Emergency Surgery 4, no. 1 (2009): 9, 10.1186/1749-7922-4-9. 19257904 PMC2660300

[2] P. M. Dion , M. Lapierre , H. Said , et al., “Rethinking Cervical Spine Clearance in Obtunded Trauma Patients: An Updated Systematic Review and Meta‐Analysis,” Injury 55, no. 3 (2024): 111308, 10.1016/j.injury.2023.111308. 38266326

[3] R. Rakosi , J. Davis , J. Miller , et al., “CT vs MRI C‐Spine Imaging for C‐Spine Clearance in Obtunded Patients in Low‐Energy Trauma Mechanisms,” American Surgeon 91, no. 8 (2025): 1341–1347, 10.1177/00031348251337146. 40249392

[4] S. A. Medeiros , D. J. Carmichael , S. N. Blakely , T. Tse , C. M. Eufemio , and M. S. Factor , “The Utility of Cervical Spine MRI in Non‐Examinable or NEXUS‐Positive Patients With Suspected Blunt Cervical Spine Trauma,” American Surgeon (2025): 31348251371195, 10.1177/00031348251371195. 40851503

[5] I. A. James , A. Moukalled , E. Yu , et al., “A Systematic Review of the Need for MRI for the Clearance of Cervical Spine Injury in Obtunded Blunt Trauma Patients After Normal Cervical Spine CT,” Journal of Emergencies, Trauma, and Shock 7, no. 4 (2014): 251–255, 10.4103/0974-2700.142611. 25400384 PMC4231259

[6] M. B. Patel , S. S. Humble , D. C. Cullinane , et al., “Cervical Spine Collar Clearance in the Obtunded Adult Blunt Trauma Patient: A Systematic Review and Practice Management Guideline From the Eastern Association for the Surgery of Trauma,” Journal of Trauma and Acute Care Surgery 78, no. 2 (2015): 430–441, 10.1097/ta.0000000000000503. 25757133 PMC4409130

[7] K. Inaba , S. Byerly , L. D. Bush , et al., “Cervical Spinal Clearance: A Prospective Western Trauma Association Multi‐Institutional Trial,” Journal of Trauma and Acute Care Surgery 81, no. 6 (2016): 1122–1130, 10.1097/ta.0000000000001194. 27438681 PMC5121083

[8] M. L. Kaiser , M. D. Whealon , C. Barrios , A. P. Kong , M. E. Lekawa , and M. O. Dolich , “The Current Role of Magnetic Resonance Imaging for Diagnosing Cervical Spine Injury in Blunt Trauma Patients With Negative Computed Tomography Scan,” American Surgeon 78, no. 10 (2012): 1156–1160, 10.1177/000313481207801032. 23025962

[9] J. Menaker , D. M. Stein , A. S. Philp , and T. M. Scalea , “40‐Slice Multidetector CT: Is MRI Still Necessary for Cervical Spine Clearance After Blunt Trauma?,” American Surgeon 76, no. 2 (2010): 157–163, 10.1177/000313481007600207. 20336892

[10] J. Menaker , A. Philp , S. Boswell , and T. M. Scalea , “Computed Tomography Alone for Cervical Spine Clearance in the Unreliable Patient‐‐Are We There Yet?,” Journal of Trauma 64, no. 4 (2008): 898–903: Discussion ‐4, 10.1097/ta.0b013e3181674675. 18404054

[11] M. Steigelman , P. Lopez , D. Dent , et al., “Screening Cervical Spine MRI After Normal Cervical Spine CT Scans in Patients in Whom Cervical Spine Injury Cannot Be Excluded by Physical Examination,” Americas Journal of Surgery 196, no. 6 (2008): 857–862: Discussion 62‐3, 10.1016/j.amjsurg.2008.07.040. 19095100

[12] J. R. Hoffman , W. R. Mower , A. B. Wolfson , K. H. Todd , and M. I. Zucker , “Validity of a Set of Clinical Criteria to Rule Out Injury to the Cervical Spine in Patients With Blunt Trauma. National Emergency X‐Radiography Utilization Study Group,” New England Journal of Medicine 343, no. 2 (2000): 94–99, 10.1056/nejm200007133430203. 10891516

[13] A. H. Kaji , D. Schriger , and S. Green , “Looking Through the Retrospectoscope: Reducing Bias in Emergency Medicine Chart Review Studies,” Annals of Emergency Medicine 64, no. 3 (2014): 292–298, 10.1016/j.annemergmed.2014.03.025. 24746846

[14] M. Raza , S. Elkhodair , A. Zaheer , and S. Yousaf , “Safe Cervical Spine Clearance in Adult Obtunded Blunt Trauma Patients on the Basis of a Normal Multidetector CT Scan‐‐A Meta‐Analysis and Cohort Study,” Injury 44, no. 11 (2013): 1589–1595, 10.1016/j.injury.2013.06.005. 23856632

[15] J. J. Como , W. H. Leukhardt , J. S. Anderson , P. A. Wilczewski , H. Samia , and J. A. Claridge , “Computed Tomography Alone May Clear the Cervical Spine in Obtunded Blunt Trauma Patients: A Prospective Evaluation of a Revised Protocol,” Journal of Trauma 70, no. 2 (2011): 345–349: Discussion 9‐51, 10.1097/ta.0b013e3182095b3c. 21307733

[16] A. E. Ertel , B. R. Robinson , and M. H. Eckman , “Cost‐Effectiveness of Cervical Spine Clearance Interventions With Litigation and Long‐Term‐Care Implications in Obtunded Adult Patients Following Blunt Injury,” Journal of Trauma and Acute Care Surgery 81, no. 5 (2016): 897–904, 10.1097/ta.0000000000001243. 27602907

[17] J. H. Badhiwala , C. K. Lai , W. Alhazzani , et al., “Cervical Spine Clearance in Obtunded Patients After Blunt Traumatic Injury: A Systematic Review,” Annals of Internal Medicine 162, no. 6 (2015): 429–437, 10.7326/m14-2351. 25775316

[18] D. Hennessy , S. Widder , D. Zygun , R. J. Hurlbert , P. Burrowes , and J. B. Kortbeek , “Cervical Spine Clearance in Obtunded Blunt Trauma Patients: A Prospective Study,” Journal of Trauma 68, no. 3 (2010): 576–582, 10.1097/ta.0b013e3181cf7e55. 20220418

[19] P. A. Anderson , Z. Gugala , R. W. Lindsey , A. J. Schoenfeld , and M. B. Harris , “Clearing the Cervical Spine in the Blunt Trauma Patient,” Journal of the American Academy of Orthopaedic Surgeons 18, no. 3 (2010): 149–159, 10.5435/00124635-201003000-00004. 20190105

[20] J. S. Smith , “A Synthesis of Research Examining Timely Removal of Cervical Collars in the Obtunded Trauma Patient With Negative Computed Tomography: An Evidence‐Based Review,” Journal of Trauma Nursing 21, no. 2 (2014): 63–67, 10.1097/jtn.0000000000000033. 24614295

[21] T. J. Harris , C. C. Blackmore , S. K. Mirza , and G. J. Jurkovich , “Clearing the Cervical Spine in Obtunded Patients,” Spine 33, no. 14 (2008): 1547–1553, 10.1097/brs.0b013e31817926c1. 18552669

[22] L. A. Tan , M. K. Kasliwal , and V. C. Traynelis , “Comparison of CT and MRI Findings for Cervical Spine Clearance in Obtunded Patients Without High Impact Trauma,” Clinical Neurology and Neurosurgery 120 (2014): 23–26, 10.1016/j.clineuro.2014.02.006. 24731570

[23] T. P. Plackett , F. Wright , A. J. Baldea , et al., “Cervical Spine Clearance When Unable to Be Cleared Clinically: A Pooled Analysis of Combined Computed Tomography and Magnetic Resonance Imaging,” Americas Journal of Surgery 211, no. 1 (2016): 115–121, 10.1016/j.amjsurg.2014.12.041. 25997715

[24] J. G. Meara , A. J. M. Leather , L. Hagander , et al., “Global Surgery 2030: Evidence and Solutions for Achieving Health, Welfare, and Economic Development,” Lancet 386, no. 9993 (2015): 569–624, 10.1016/s0140-6736(15)60160-x. 25924834

